# A stress-reduction eHealth intervention for healthcare workers in primary care settings: an implementation study

**DOI:** 10.3389/fpubh.2025.1600059

**Published:** 2025-05-21

**Authors:** Blanca Garcia-Vazquez, Ainoa Muñoz-Sanjosé, Andrea Fernández-López, Irene Pérez-de-Ciriza, Luis Nocete-Navarro, Elena Almaraz-Garzón, María Teresa Martín-Palacios, Blanca Novella, María Fe Bravo-Ortiz, José Luis Ayuso-Mateos, Carmen Bayón, Roberto Mediavilla

**Affiliations:** ^1^Department of Psychiatry, Clinical Psychology and Mental Health, Hospital Universitario La Paz, Madrid, Spain; ^2^Department of Psychiatry, Universidad Autónoma de Madrid, Madrid, Spain; ^3^Instituto de Investigación Sanitaria del Hospital Universitario de La Paz (IdiPAZ), Madrid, Spain; ^4^Centro de Investigación Biomédica en Red de Salud Mental (CIBERSAM), Instituto de Salud Carlos III, Madrid, Spain; ^5^Department of Psychiatry, Hospital Universitario La Princesa, Madrid, Spain; ^6^Bustarviejo Primary Care Health Centre, Madrid, Spain; ^7^Instituto de Investigación Sanitaria del Hospital Universitario La Princesa, Madrid, Spain; ^8^Area of Technological Evaluation and Health Innovation, Dirección General Asistencial, Madrid, Spain; ^9^Centro de Salud Potosí, Gerencia de Atención Primaria, Madrid, Spain

**Keywords:** mental health, healthcare workers, eHealth, implementation science, public health, occupational health

## Abstract

**Introduction:**

Healthcare systems across Europe are facing significant challenges in retaining and recruiting healthcare workers (HCWs). Mental health problems, including anxiety, depression, and burnout, are major drivers of turnover. Although some psychological interventions, particularly eHealth tools, are effective, they are rarely tested under real-world conditions, widening the research-implementation gap. This study evaluates the implementation outcomes of an eHealth intervention that was shown to reduce anxiety and depression among frontline HCWs during the COVID-19 pandemic.

**Methods:**

The study was conducted at a primary care centre affiliated with Hospital Universitario La Paz in Madrid, between October 2023 and February 2024. The intervention “Doing What Matters in Times of Stress” (DWM), consisted of a web-based, self-help tool and was offered in hybrid and remote formats. Mixed methods were employed to assess key implementation outcomes from Proctor’s framework, combining quantitative data from pre- and postintervention assessments with qualitative insights from interviews.

**Results:**

Seventeen participants were included in the study, with 59% choosing the hybrid format and 41% selecting remote sessions. Participation rate was 44% and retention rates were 80 and 100% for the hybrid and remote formats, respectively. The intervention was perceived as acceptable, appropriate, and feasible. Flexible delivery formats and robust group dynamics, particularly in the hybrid format, were identified as key contributors to the intervention’s success, enhancing group cohesion and fostering empathy among participants. Barriers, such as stigma and time constraints were identified, while enabling factors included practical content and flexibility.

**Conclusion:**

This study evaluated the fidelity, feasibility, acceptability, and appropriateness of DWM intervention and remote/hybrid delivery formats among HCWs at a primary care centre in Madrid. Data suggests future studies should maintain hybrid and remote delivery formats and address specific access challenges. These findings provide crucial insights for expanding mental health interventions for HCWs across diverse settings, with implications for public health policy.

## Introduction

Europe is facing a significant healthcare workforce crisis (1). Current trends suggest that the shortage of healthcare workers (HCWs) in the European Union will triple in the coming years, with an expected deficit of 4 million by 2030. Retaining and recruiting HCWs is becoming increasingly difficult due to the rising demand, driven by a growing population and longer life expectancy across the continent (2).

Psychological conditions significantly contribute to the increased turnover among doctors and nurses (3, 4). After COVID-19, HCWs reported higher levels of burnout, demanding work conditions, and general dissatisfaction with their roles (1, 5). Recent studies estimate that one in four HCWs experiences symptoms compatible with anxiety and/or depression (6–8). These symptoms are associated with turnover intention and absenteeism among health professionals (5, 9). This establishes a feedback loop in which mental health issues and workforce shortages continually exacerbate each other.

There are mental health interventions that have shown promising effects, but they rarely translate into public health policies and occupational strategies (10, 11). For example, there are effective group treatments that focus on emotional regulation skills (12), as well as single-session interventions with positive short-term outcomes (13, 14). Most studies conducted after the COVID-19 outbreak used eHealth interventions, due to lockdowns and movement restrictions. For example, a Randomised Controlled Trial (RCT) in England reported a reduction in psychiatric symptoms among HCWs after using a smartphone-based intervention (15). Similarly, in Singapore, mindfulness practice through a smartphone app reduced distress and improved psychological well-being in HCWs (16). In the EU, two interventions developed by the World Health Organization (WHO) were combined in an eHealth, stepped-care programme as part of the project entitled *Preparedness of health systems to reduce mental health and psychosocial concerns resulting from the COVID-19 pandemic* (RESPOND). The interventions were a stress management course included in Self Help Plus (SH+) called Doing What Matters in Times of Stress (DWM) (17), and a brief intervention based on cognitive–behavioural and problem-solving strategies called Problem Management Plus (PM+) (18). A multicentre trial in Spain demonstrated the effectiveness of this intervention in reducing anxiety and depression symptoms at a 2-month follow-up among HCWs with psychological distress (19). In addition to the effectiveness measures, a process evaluation of the RESPOND clinical trial was also performed in the Community of Madrid (20). To our knowledge, this was the only study that conducted a detailed process evaluation, analysing not only the intervention’s impact on mental health symptoms but also key implementation outcomes. The results indicated that the intervention was feasible, appropriate, and timely. Participants expressed high levels of acceptance, pointing to several factors that facilitated success, such as schedule flexibility, positive relationships with facilitators, and specific intervention components. These findings informed the design and development of the present implementation study.

This study aimed to implement the DWM intervention among healthcare workers at a primary care centre within the Madrilenian Health Service (SERMAS) in Spain. The intervention was adapted based on the previous trial evaluation (20), incorporating the socioecological model (21) and findings from other RESPOND trials and similar interventions (22). We built upon the implementation protocol included in the European Commission’s Best Practices Portal. Key implementation outcomes were assessed following Proctor’s model (23), while barriers and facilitators specific to the setting were also identified.

## Materials and methods

### Setting and study design

The study took place at a primary care centre in the northern part of the city of Madrid (Spain), which is associated with a catchment area of more than 20.000 people. Data collection started in October 2023 and finished in February 2024. The Ethics Committee at Hospital Universitario La Paz approved the project (identifier 2023.635). All the participants signed the informed consent form and none were compensated for participation. In addition, within the hybrid format, all participants agreed from the first group session not to disclose any information discussed during the meetings. Findings follow the Standards for Reporting Implementation Studies (StaRI) guidelines (24). The checklist is available in the Supplementary material.

### Participants recruitment

The target population consisted of healthcare workers (HCWs) directly employed by the Department of Health, which excluded cleaning and security staff. At the study onset, the centre had 13 doctors, five medical residents, 10 nurses, six administrative staff, four ancillary workers, and one social worker (*N* = 39). The median age of all centre workers was 51, and 80% were female. The participants’ ages ranged from 26 to 65 years, with a mean age of 51 years, and a 80% of females. All were invited to participate as described below. There were no exclusion or withdrawal criteria.

### Intervention and implementation strategies

DWM is a guided self-help web application consisting of five weekly modules that incorporate strategies based on Acceptance and Commitment Therapy (ACT), mindfulness techniques, and audio recordings for practice. It is part of SH+ (17) and was adapted to HCWs in a digital format as part of the initial procedures of the EU-funded project RESPOND (25). In the current study, we offered this HCW-adapted version of DWM in remote and hybrid formats, allowing participants to choose based on their preferences. The remote format included a web-based self-help guide and weekly 15-min support calls. The hybrid format included a web-based self-help guide and weekly 45-min in-person group sessions, that took place during the transition between morning and afternoon shifts to facilitate attendance. The intervention providers were mental health professionals in training (clinical psychology and mental health nursing) who received specific preparation (~50 h) and attended supervision sessions while the trial was ongoing (~10 h). The trainers and supervisor were psychiatrists and clinical psychologists instructed by the intervention developers.

**Figure 1:**
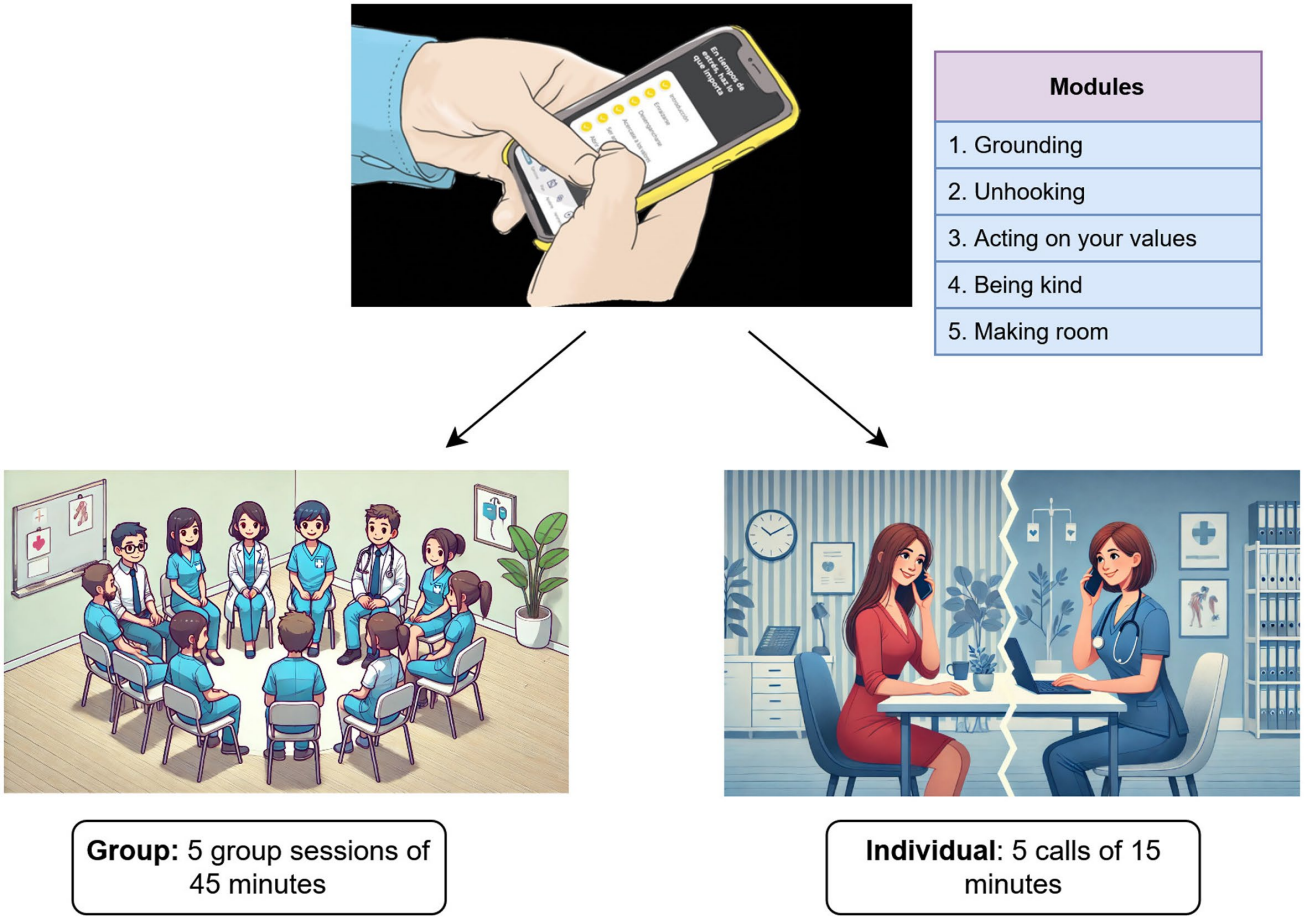
Doing What Matters (DWM) contents and delivery formats.

All were involved as providers and supervisors in the previous RESPOND trial, ensuring continuity and familiarity with the intervention protocol. Mental health nurses provided phone support, and in-person sessions were led by a clinical psychology trainee working at the centre. All intervention providers received training to deliver the interventions and were supervised by a psychiatrist qualified as a DWM trainer. Further details regarding the DWM intervention, training, and supervision are available elsewhere (25). Intervention modules and the implementation formats are shown in Figure 1. Adapted content for group sessions is available in the Supplementary material.

We employed several implementation strategies to adapt the DWM intervention in our specific setting. These strategies were guided by the Expert Recommendations for Implementing Change (ERIC) project (26). Table 1 outlines the strategies used and how they were applied across different phases of the study: implementation planning, pre-intervention, intervention, and post-intervention data collection (Table 1).

**Table 1:** Implementation strategies developed in each phase of the implementation study.

Phase	Implementation strategy from ERIC project	Application in our study
Implementation planning	Build a coalition	Intervention providers were familiar with each other and the supervisor due to their collaboration in the previous trial
	Capture and share local knowledge	Implementers from other countries provided advice and guidance to the research team
	Conduct local needs assessment	The process evaluation from the previous trial informed the design and execution of this implementation study
	Develop academic partnerships	The study was conducted as part of a PhD project, led by a dedicated research team
	Develop and organize quality monitoring systems	Scheduled supervision meetings were held, and facilitators’ activities were systematically recorded
	Promote adaptability	The process evaluation from the previous trial identified the active ingredients of the DWM programme
	Tailor strategies	Based on insights from the prior trial, only the DWM programme was applied, with group sessions offered as an alternative to individual calls
Pre-intervention	Conduct educational meetings	An introductory meeting was organised to present the study, the intervention, and the delivery formats to participants
	Inform local opinion leaders	The centre’s manager was actively involved as part of the research team
Intervention	Change service sites	The in-person group format was delivered at the participants’ workplace to ensure accessibility
	Distribute educational materials	The intervention was guided, with theoretical and practical materials available through an app or website
	Intervene with consumers to enhance uptake and adherence	During calls and group sessions, facilitators encouraged participants to practice regularly and utilise the app as much as possible
	Promote network weaving	The hybrid delivery format allowed participants to share experiences. Facilitators encouraged participants to apply and teach DWM tools to their families and patients
	Provide clinical supervision	Facilitators attended two scheduled supervision meetings to address any challenges related to DWM delivery
Post-intervention	Revise professional roles	Stakeholders and participants were asked about their perspectives on the role of the intervention providers
	Purposely reexamine implementation	Both implementation outcomes and barriers and facilitators were measured in the study

### Procedure

Prior to data collection, we reached out to the manager to confirm their collaboration. In October 2023, we organized an informational session during the overlap of the morning and afternoon shifts to optimize attendance. To promote the session, the centre’s director sent an institutional email, and flyers were posted in break areas. During the meeting, we provided a brief overview of each module and explained the remote and hybrid delivery formats. By mid-November, participant recruitment was completed, and pre-intervention questionnaires were administered.

The intervention was delivered from November 13^th^ to December 18^th^, 2023. There were two supervision meetings with a RESPOND supervisor during this period. In the third week of December, post-intervention questionnaires and interviews were conducted by a team member not involved in the intervention to minimize bias. In-depth interviews were also conducted with intervention providers and key stakeholders, such as mental health managers, hospital administrators, and primary care centre directors, to explore their perspectives on the intervention and its feasibility within the context. These interviews were completed in January and February 2024. The scripts of the interviews are available in the Supplementary material.

### Data collection and data analysis

Collected data included both quantitative and qualitative information. Quantitative data encompassed sociodemographic variables (age, gender and type of job), as well as clinical information; psychological distress was assessed using the Kessler Psychological Distress Scale (K-10), with a score of ≥16 indicating distress (27). Qualitative insights focused on key implementation outcomes based on Proctor’s model (23), including acceptability, appropriateness, feasibility, fidelity, and recommendations for future large-scale implementation. Table 2 details the sources used for information related to these outcomes. Quantitative data were obtained from pre- and post-evaluations, whereas qualitative data came from semi-structured interviews with participants and key informants. We also conducted a group semi-structured interview with the intervention providers. Questionnaires and interview scripts are available in the Supplementary material. Participants who did not complete the modules on the app were classified as dropouts, and those who finished the intervention but did not participate in the post-evaluation were considered lost to follow-up.

**Table 2:** Data sources and methods used to collect information related to implementation outcomes.

Outcome	Source	Methods		Description
		Qualitative data	Quantitative data	
Acceptability-satisfaction	Monitoring data		Administrative data	Recruitment and retention rates. (incl. dropouts in each format)
	Intervention providers	Implementers interview		Deliverers were asked about participants’ format preferences and the delivery formats applied
	Trial participants	Participants interviews	Satisfaction rating	Intervention users provided feedback on their format choices, the chosen delivery format, and the peer support received. Satisfaction was rated on a 0–10 scale
Appropriateness-usefulness	Decision makers	Stakeholder interviews		Decision-makers were consulted on whether the programme was appropriate, timely, and compatible with the context
	Trial participants	Participants interviews		Intervention users were asked about the usefulness of the programme content and its compatibility with existing mental health resources
	Intervention providers	Implementers interview		Intervention providers were asked whether the programme was appropriate, timely, and relevant
Feasibility	Monitoring data		Administrative data	Attrition rates for follow-up measures were documented
	Intervention providers	Implementers interviews		Deliverers were asked for their opinions on the suitability of having professionals in training act as facilitators
	Decision makers	Stakeholder interviews		Decision-makers were asked about the training provided to facilitators and the resources needed for future implementation
Fidelity	Monitoring data (hybrid format)		Implementer self-report	Attendance at group sessions was recorded, as well as deviations from the planned timing and activities
	Monitoring data (remote format)		Implementer self-report	Deviations from the expected frequency and duration of calls were also noted

We used mixed methods to analyse data from multiple sources. Quantitative data were described using statistics such as means, frequencies and percentages. Qualitative data were collected through interviews, and recordings were transcribed using an automated tool (NVivo 11—Transcription). A single coder analysed the transcripts using thematic analysis based on Braun and Clarke’s guidelines (28) with Proctor’s implementation outcomes as the coding frame (23). The coding scheme was informed by the sources listed in Table 2, corresponding to each implementation outcome. Representative quotes were extracted to summarize the key findings for each outcome. Another author independently reviewed the analysis to resolve discrepancies and ensure accuracy in the final results. The analysis also revealed specific barriers and facilitators relevant to the intervention context. Our approach to data analysis was deductive and essentialist/realist, and the level of content was explicit (i.e., semantic).

## Results

### Characteristics of the implementation process

Seventeen participants were included between October and December 2023 (participation rate: 17/39, 44%). Of these, 14 were female (82%), and 10 chose the hybrid format, meaning that 59% of the participants preferred to attend in-person group sessions, whereas 41% (seven participants) opted for individual remote sessions. The sample had a mean age of 43 years. All participants had university studies and half had rotating shift schedules (47%). Of the 17 professionals enrolled in the study, 15 completed the intervention, while only 12 (70%) completed the follow-up assessments. Two participants dropped out during the programme, and three were lost to follow-up. The dropouts were both in the hybrid format, thus, retention rates were 100 and 80% for the remote and hybrid formats, respectively. Further sociodemographic and administrative information from each format is shown in Table 3.

**Table 3:** Sociodemographic information during the implementation process.

	Total participants (*N* = 17)	Remote format (*N* = 7)	Hybrid format (*N* = 10)
Age in years, Median	43	42	44
Gender [female], *n* (%)	14 (82%)	8 (80%)	6 (85%)
Job type			
Physician	13	5	9
Nurse	3	2	1
Other	1	0	1
Satisfaction (0–10)	8	8	9
Dropouts	2	0	2

In the initial evaluation, 88% of the participants (15/17) reported symptoms consistent with psychological distress, as measured by the K-10. After the intervention, this percentage was 66% (8/12). Specifically, of the 12 participants who completed the follow-up assessments, three moved from distress to no distress, and none showed the opposite trend. The rest remained above (*n* = 8) or below (*n* = 1) the threshold after the intervention.

### Implementation outcomes

Table 4 provides an overview of the results related to the implementation outcomes, which are described in detail throughout this section.

**Table 4:** Summary of results for implementation outcomes.

Outcome	Results
Acceptability-satisfaction	Both delivery formats were well accepted by users and stakeholders, each offering specific advantages. Moreover, allowing participants to choose their preferred format was viewed positively, as it contributed to increased engagement.
Appropriateness-usefulness	The intervention addressed current mental health needs and was considered well-suited for integration into daily routines. Its content was viewed as relevant to the concerns of healthcare workers, and particularly necessary in emergency and primary care settings.
Feasibility	The intervention was delivered as planned and proved compatible with the routine duties of the providers. Programme promotion and recruitment strategies were effective. The role of intervention provider appeared to be appropriate for medical and nursing residents.
Fidelity	High fidelity was achieved; sessions followed planned duration and content. Calls lasted ~15 min with near-full attendance; hybrid sessions had a median of 8 participants (45–50 min).

#### Acceptability

Both participants and stakeholders considered the online delivery of the intervention, via an app or website, as a significant advantage. The flexibility to choose between a hybrid or fully remote format, based on individual preferences and scheduling constraints, was also highly valued.

*Well, I think it’s fantastic. For me, it worked out perfectly. Otherwise, I would not have been able to take part, and I would’ve really regretted missing out. So, I think having both in-person and phone options is absolutely essential. You’ve got to keep that for next time. Honestly, the phone option did not feel distant to me at all—it actually felt very personal* (TP 5).

Participants in the hybrid format emphasized several advantages, including increased motivation to practice, stronger group cohesion, and the convenience of coordinating and attending in-person group sessions. In contrast, those in the remote format appreciated individual attention, which they felt allowed more openness with the facilitator. Overall, both approaches were satisfactory to users.

*In the group meetings, you see how everyone handles things a bit differently. We’re already outside the work environment, but at the same time, everyone has their own struggles. Maybe the person who’s always smiling does not actually know how to cope with anything. And that’s when you realise, through those moments, how we all share the techniques each of us uses* (TP 1).

Consistent with findings from previous RCTs (19), the fact that the intervention was delivered by trainee clinical psychologists, was not perceived as a limitation. Rather, it was perceived positively, as it fostered emotional openness and closer connections.

#### Appropriateness

All participants agreed that the intervention was timely and well-suited for integration into their daily routines. Interviewees indicated that the module content was highly relevant to the challenges faced by healthcare workers and addressed most of their needs. They particularly noted the value and novelty of the module about values and kindness. Additionally, mindfulness exercises and content related to unhooking skills were highlighted as especially useful.

*Anxiety and things like that. I mean, I think it’s an app that could be incredibly useful. For the general population, it would undoubtedly be highly beneficial. In fact, I’ve already mentioned some aspects because I talk a lot with my patients. We were discussing the project, and many patients, even younger ones aged between 20 and 50, said it would genuinely help them. I’ve seen elements in it that could be very valuable* (TP 12).

Although the COVID-19 emergency is no longer prevalent, the intervention providers deemed it timely. Both stakeholders and participants agreed that there remains a significant need for mental health support among healthcare workers in Madrid. Both participants and facilitators believed that the intervention was beneficial to the intended audience.

*I believe so. And I think it’s more useful for them now than it was during the pandemic. During the pandemic, I did not see them engaged at all. They seemed to take it more seriously in the second phase. Now they are in a better place, and this is helping them* (IP 1).

When asked about future interventions, some stakeholders specifically highlighted a need for psychological support in Emergency and Primary Care settings. Participants also perceived the intervention as compatible with, and complementary to existing resources.

*It’s not directly related, no, but it’s compatible—or it could be. You see, in those centres, they see you every two or three weeks. This, though, feels much closer. I feel much more empathy with you, or even with my assistant over the phone, than with the psychiatrist, who always seems to keep a certain distance. There’s a barrier* (TP 2).

#### Feasibility

Regarding the delivery format, both stakeholders and intervention providers reported that they were able to conduct weekly calls or group sessions alongside their regular jobs. The participants also found the recruiting strategies and the intervention providers to be adequate. Stakeholders emphasized the importance of involving the centre’s management team in promoting the intervention and encouraging participation. They found it effective to introduce the programme through a meeting and to notify potential participants via institutional email, informal phone messages, and posters.

*I think it’s important to have a meeting where everyone is present, and the centre’s management actively promotes it. This way, you can explain that it’s meant for everyone and will address issues that concern everyone* (DM 5).

Concerning potential training for future facilitators, stakeholders and intervention providers found it to be both interesting and beneficial, particularly for hospital residents, especially mental health nurses, but also for other medical specialists. They suggested that current training sessions offer a suitable platform for preparing new implementers.

*Of course, it’s useful—I believe it applies to any professional profile. There’s clearly a general deficit in everything related to the human aspects, communication, and practical tools. This is evident, as we receive very little training in these areas, whether at the undergraduate level or as specialists. Some specialties do include it, but it’s very limited overall* (DM2).

In terms of large-scale implementation, both participants and decision-makers believe there will be broad interest and acceptance among healthcare workers and stakeholders in mid-level positions, such as Primary Care Centre Directors or Heads of Services.

#### Fidelity

It was examined through two key dimensions: participants’ actual exposure to the intervention and implementers’ adherence to the intervention protocol.

Regarding the first aspect, self-reported data indicated that, in the remote format, calls lasted approximately 15 min. All participants attended their scheduled calls except one, who received follow-up messages. In the hybrid format, the majority of participants attended at least four group sessions, with 70% of individuals participating in nearly 70% of the scheduled meetings. Each session lasted between 45 and 50 min. All the content was covered, and the sessions remained close to the expected time. Across all participants, digital usage records from the website indicated that nearly all individuals completed all available modules and lessons (a median of 6 modules and 21 lessons, representing the maximum content). The median number of logins was 13 (ranging from 2 to 42), and the median time spent on the website was 2 min, with a range from 1 min to a maximum of 8 h.

About the second aspect, no independent fidelity assessments were conducted within the current study. However, in a previous study involving the same intervention providers and supervisor, independent fidelity assessments of recorded calls were performed using structured observation protocols. These assessments, carried out by supervisors, demonstrated high fidelity levels, ranging from 90 to 100% (20).

### Context

We identified several specific barriers and facilitators for Primary Care Centres within the Madrilenian Public Health Service.

#### Barriers

Participants and stakeholders noted barriers associated with the hybrid format, particularly in terms of group participation. These barriers included reluctance to openly discuss mental health issues and the risk of low engagement in centres with strained staff relationships. The implementer also noted challenges stemming from their dual role as a resident in the same centre, leading to role ambiguity, although this did not appear to affect the intervention itself. Interestingly, participants did not express any concerns regarding peer support.

Regarding the intervention formats, participants identified additional barriers such as limited awareness of mental health issues, a lack of confidence in the effectiveness of the treatment, and insufficient time to engage in practice.

*Well, many people do not really believe in these things. Even among us, healthcare workers, you talk to some, and all you hear is dismissive comments—it’s always the same. It’s like, “What’s the point? We do not even have time for that, so why bother?” Yes, even within our profession, there’s a lack of time and, as I mentioned, a lack of faith in its usefulness* (TP 5).

For future interventions, stakeholders expressed concerns about the willingness of politicians. They also observed a reluctance among politicians to acknowledge workers’ distress, as this might impact the institution’s reputation.

*And secondly, it might also be that, in the end, this involves discussing something that, in a way, reflects poorly on the organisation, do not you think?* (DM 2).

The reasons why workers who chose not to participate were also explored anonymously. Three individuals cited a lack of time, one mentioned not needing psychological support, and another believed that the content was not suitable for administrative staff.

#### Facilitators

Both participants and stakeholders agreed on the benefits of allowing individuals to select their preferred application format. In the hybrid format, group sessions fostered empathy and enhanced interpersonal relationships among team members. Additionally, motivation towards the practice increased weekly, according to group follow-up assessments and the implementer’s point of view.

*And then, seeing that the person next to them was practising and it was working, they started practising too, and in the end, everyone was practising* (IP 3).

In terms of the intervention itself, participants noted that prior experience with meditation techniques facilitated their engagement with the practices. They also expressed high satisfaction with the simplicity and practicality of the content, the accessibility of the audio materials, and the ease of weekly follow-ups, which benefited from flexible scheduling.

*But you have to attend. So, offering it this way seemed very convenient and accessible to me. I participated with a lot of interest* (TP 2).

The facilitators found this trial to be simpler than the previous one, which was attributed to the participants having fewer symptoms and their own increased experience in their roles.

## Discussion

### Main results

We conducted an implementation study of the eHealth intervention “Doing What Matters in Times of Stress” (DWM) for healthcare workers (HCWs) at a primary care centre in Madrid. The intervention was delivered in both remote and hybrid formats. Results showed that the intervention was feasible and appropriate, with participants finding the implementation strategies to be adequate, acceptable, and well-received. Identified barriers included stigma, low mental health awareness, and limited time for practice and attendance. Enabling factors were format choice, simple and practical content, and increased motivation from group sessions. These insights can guide future adjustments to delivery strategies and content.

### Implementation outcomes

The intervention was well-accepted, as demonstrated by the participation of nearly half of the centre’s staff, which contrasts with the generally low level of engagement in mental health resources previously reported among HCWs (13–33% receiving psychological support during COVID-19 pandemic) (29, 30). A key factor contributing to this greater degree of participation was the flexibility offered. First, the programme was open to all healthcare professionals at the centre, without requiring psychological distress or diagnostic screening. Instead, the intervention was presented as a guided self-help tool for stress management, potentially reducing internalised mental health stigma commonly reported among HCWs (31–33) and encouraging engagement. Second, participants could select either a fully remote or hybrid delivery format, allowing them to choose what best suited their schedules and preferences. Additionally, the study was coordinated by a temporary staff member who addressed participants’ questions, both before and during the intervention, further supporting engagement. The high acceptability rates observed in this study are particularly encouraging. However, it is noteworthy that none of the administrative or ancillary staff chose to participate (representing 28% of the centre’s workforce). Future studies might focus on non-participants, exploring drop-out reasons and informing adaptations that make the intervention more accessible to them.

The intervention was also considered appropriate. On the one hand, participants were generally satisfied with the content. They saw DWM as a simple and applicable tool and were particularly keen on some of its elements –namely the modules of personal values and kindness, or mindfulness practices. On the other hand, nearly one in four participants reported a reduction in distress rates, which aligns with previous research showing DWM’s effectiveness in reducing anxiety and depression symptoms (19). However, given the study design, we cannot draw conclusions about the intervention’s effects.

The results are promising regarding the feasibility of a larger-scale implementation in the Madrilenian Health Service (SERMAS). Notably, there were very few dropouts—one participant left due to lack of interest, and another for personal reasons unrelated to the study. Both the facilitators and the participants reported that the intervention could be easily integrated into their routines. Furthermore, residents appeared well-suited to serve as facilitators, a promising finding for future trials. This aligns with current research underlining the value of peer support in mental health interventions (34, 35), as well as recent health recommendations, like the guidelines developed by the Mental Health Commission of Canada (36). Additionally, this intervention could be used as a preventive tool that is coordinated with more specific resources, such as the Plan for Sick Healthcare Workers (PAIPSE) programme in Madrid. Future implementation studies may align with WHO roadmaps and examine how peer-supported strategies (37) might complement existing mental health support tools for HCWs.

Finally, DWM was delivered in alignment with its implementation manuals. The intervention’s highly standardized structure previously contributed to high adherence to remote implementation protocols, with fidelity levels ranging from 90 to 100% in the earlier trial (20). In this study, both remote and hybrid formats also demonstrated acceptable adherence levels, suggesting that similar initiatives could be scaled up effectively. Integrating DWM with other WHO initiatives, such as EQUIP, could further support the consistent delivery of care across various settings (38).

### Barriers and enabling factors in primary care settings in Madrid

We identified specific barriers and facilitators impacting the implementation of mental health interventions for HCWs in primary care centres in Madrid. Consistent with prior research indicating HCWs’ difficulties in accessing psychological support (31, 32), we observed barriers such as limited time, low mental health awareness, and scepticism about intervention efficacy. However, our study also highlighted several facilitators that help address these challenges. Firstly, framing the intervention as a preventive workplace tool may help reduce stigma and improve accessibility. Additionally, as an eHealth-based intervention, it offers flexibility through remote delivery, accommodating varied work schedules and potentially reaching a wider audience. Moreover, the intervention’s effectiveness is well-supported by prior research and its recognition in the European Commission’s Best Practices Portal, aligning with the broader movement for evidence-based public health strategies (39).

Another potential barrier relates to the role of intervention providers in this setting. Having implementers who are also members of the centre’s staff may pose challenges, such as role ambiguity outside the sessions. However, in line with previous data that underscore the value of peer support (34, 35), our findings suggest that a pre-existing relationship between implementers and participants positively influences the session atmosphere and fosters emotional openness, partly due to the confidentiality commitment made during the study. Some stakeholders also noted that centres with weaker interpersonal relationships might face challenges in group session participation. Interestingly, participants in our study’s hybrid format reported enhanced empathy and normalising emotions. This suggests that offering both remote and hybrid formats in future implementations may foster accessibility and engagement across varied team dynamics.

Lastly, our study observed uneven participation across professional roles within the centre, with administrative and ancillary staff notably absent from the programme. Future initiatives should be inclusive, inviting HCWs from all professional categories to participate and ensuring equal access. Conducting local qualitative studies could help to identify specific access barriers across professional groups and inform tailored engagement strategies. Large-scale effectiveness-implementation trials are necessary to evaluate the intervention’s impact and broader applicability in diverse healthcare settings, particularly in environments where mental health support is less structured.

### Limitations

We acknowledge several limitations in our study that require cautious interpretation of the findings. First, our findings may not be generalisable to all healthcare workers in Madrid, as the context and needs in primary care differ from those in hospital or emergency settings. Additionally, the implementation was conducted in only one primary care centre, highlighting the need for future studies to include multiple centres. Second, some participants who completed the intervention did not complete follow-up evaluations, which may introduce a bias favouring individuals with positive attitudes towards the intervention. Additionally, we were unable to formally interview participants who dropped out, although they informally cited personal reasons for leaving; proper interviews would have been important for assessing key implementation outcomes. Third, the sample may be biased due to the predominance of female physicians among participants, resulting in an overrepresentation of female participants and an underrepresentation of ancillary and administrative staff. This imbalance may limit the generalisability of the findings, and self-selection bias could also influence the acceptability outcomes. Future studies in larger settings should investigate potential barriers for administrative and ancillary staff to engage with the programme. Finally, as this was not a full-scale implementation trial, we cannot draw conclusions regarding adoption, cost, reach, or sustainability. Our focus was on early-stage implementation outcomes (such as acceptability, appropriateness, feasibility, and fidelity). Future research should use more robust designs to assess later-stage outcomes and explore cost-effectiveness, providing a stronger basis for public health policy decisions.

### Implications

This study has significant implications for research and policy. For research, it adds real-world evidence that builds on previous findings (19, 25). This aligns with recent recommendations in implementation science, which stresses the importance of moving beyond randomised controlled trials to assess implementation outcomes in practical settings (10, 11). To date, DWM has been shown to be both effective and acceptable for HCWs (19, 20), with successful delivery in a primary care setting. Future research should aim to replicate these findings in other settings and conduct large-scale implementation trials comparing different implementation strategies and DWM formats. However, to strengthen and generalise these recommendations, future large-scale studies should also assess the cost-effectiveness of the intervention and address the limitations identified in the present study. For instance, it would be important to examine whether the observed bias persists in larger samples, to focus on recruitment strategies targeting underrepresented professional groups such as administrative and ancillary staff, and to explore whether the intervention requires adaptations to better align with their specific needs and interests.

From a policy perspective, our results can inform strategies to address Europe’s healthcare workforce crisis (1). This intervention is effective, highly scalable, and adaptable to multiple formats in real-world conditions. To optimise the use of limited resources and support future scalability, decision-makers should consider the strategies suggested by our interviewees, such as focusing on centres facing the highest demand, using trainees as facilitators, considering follow-up reminders or group sessions post-intervention, and coordinating with mental health services when needed. Ensuring long-term sustainability will require transferring ownership to local actors to enhance organisational adoption in future large-scale implementations. While political will and financial constraints remain key challenges, many of the barriers identified in this pilot study could be mitigated through the proposed measures. Additionally, while the program may address some inherent challenges of the healthcare profession (e.g., night shifts, unexpected demands, or exposure to trauma), it is equally important for decision-makers to address modifiable factors impacting mental health (e.g., salary, breaks, and adequate equipment). Since barriers and facilitators may differ across settings and over time, we recommend conducting local needs assessments before launching large-scale studies, followed by periodic evaluations.

## Conclusion

Policymakers in Europe must focus on retaining HCWs amid increasingly challenging conditions by promoting mental health and well-being in the workplace. Findings from this implementation study demonstrate that a highly manualized eHealth intervention can be successfully adapted and implemented with high acceptability in real-world conditions in Spain. A detailed guide for tailoring, adapting, and implementing the intervention in other settings is available on the European Commission’s best practices portal and should be used by decision-makers as a tool to complement broader organizational policies.

## Data Availability

Anonymised raw data supporting the conclusions of this article will be made available by the authors upon reasonable request, in full compliance with the General Data Protection Regulation (GDPR) and without undue reservation.
