# Supplementary material for: A stress-reduction eHealth intervention for healthcare workers in primary care settings: an implementation study
Source: Front Public Health. 2025 May 21;13:1600059. doi: 10.3389/fpubh.2025.1600059 (PMC12149415; doi:10.3389/fpubh.2025.1600059)
Supplement: Supplementary file 1 [file Table_1.docx]

***Supplementary material***

**Supplementary tables**

1. **DWM group sessions**

| **Time** | **Description** |
| --- | --- |
| **Module 1: Grounding** | |
| 10 minutes | Welcome and course overview: introduction of facilitator and participants.  Overview of course modules and guidelines: confidentiality, respect, using “I” statements, avoiding advice. |
| 10 minutes | Stress and “getting hooked”: group discussion about stress and common triggers. |
| 10 minutes | Examples of mindfulness: hare examples in pairs, then discuss as a group. |
| 10 minutes | Concept of “emotional storm” and grounding exercise: explore the in small groups, then debrief in the larger group. |
| 5 minutes | Closeness and next steps: summarize learnings, encourage continued practice, and promote Module 2. |
| **Module 2: Unhooking** | |
| 5 minutes | Welcome: refresh key points from Module 1. |
| 10 minutes | Concepts and practice discussion: explore the session’s concepts in pairs and as a group. |
| 15 minutes | Revisiting stress and unhooking concepts: explain the interaction between thoughts, emotions, and sensations. Practice naming and unhooking from difficult feelings. |
| 10 minutes | Grounding practice and sharing: brief grounding activity followed by personal reflections. |
| 5 minutes | Closeness and next steps: summarize insights, encourage practice, and promote Module 3. |
| **Module 3: Acting on Your Values** | |
| 5 minutes | Welcome and review: recap previous modules and set the stage for values-focused activities. |
| 10 minutes | Unhooking and grounding challenges: discuss difficulties in small groups and with the whole group. |
| 15 minutes | Exploring values: identify personal values through individual and pair exercises. |
| 10 minutes | Action plan: create and share a personal plan to act on values over the coming week. |
| 5 minutes | Closeness and next steps: reinforce action plans, encourage ongoing practice, and promote Module 4. |
| **Module 4: Being Kind** | |
| 5 minutes | Welcome and concept: highlight nearing course completion and link with previous modules. |
| 10 minutes | Review and challenges: discuss difficulties with the action plan and practice as a group. |
| 15 minutes | Kindness exercises: focus on unhooking from self-criticism. Practice kindness towards others and oneself. |
| 10 minutes | Problem solving: use personal values to guide solutions in pairs, then discuss as a group. |
| 5 minutes | Closeness and next steps: summarize the session and encourage enrollment in Module 5. |
| **Module 5: Making Room** | |
| 5 minutes | Welcome and recap: acknowledge course completion and review prior modules. |
| 15 minutes | Review and group discussion: reflect on practices from the week in small groups. Revisit problem-solving challenges as a large group. |
| 15 minutes | Making room for discomfort: Share personal strategies for managing discomfort. Discuss short- and long-term impacts of these strategies. |
| 10 minutes | Closing with gratitude: reflect on course practices, emphasize ongoing application, and express gratitude. |

1. **Interview Scripts**

***Interview script: trial participants***

**Baseline assessment**

1. Age (in years)

2. Gender

a. Male

b. Female

c. Other gender

3. What is your highest completed level of education?

a. Incomplete primary schooling

b. Primary school

c. Secondary school

d. Technical-professional training

e. Undergraduate degree (university training)

f. Postgraduate studies (master's, doctorate, medical specialty, etc.)

4. What is your current job?

a. Physician

b. Nurse

c. Ancillary worker

d. Ocuppational worker

e. Social worker

f. Administrative

g. Other

5. Do you have Schedule shifts?

a. Yes

b. No

6. Have you got rotating shifts?

a. Yes

b. No

7. Do you currently receive online or face-to-face psychological support?

a. Yes (specify)

b. No

8. Do you currently any psychiatric mediation?

a. Yes (specify)

b. No

9. Have you got any mental health diagnosis?

a. Yes (specify)

b. No

10. Kessler distress scale (K-10)

*In the past month…*

******

**Follow-up assessment:**

11. Do you think the intervention you have received is timely for the psychological needs of the HCWs?

a. Yes

b. No, why?

12. Do you think is possible to complete the intervention in a proper way as part of your daily routine?

a. Yes

b. No, why?

13. From 0 to 10, to what extent are you satisfied with the intervention received?

14. Kessler Distress Scale (K-10)

*In the past month…*

**Interview:**

**Introduction**

• Greet the person. Introduce yourself

• Remember the participant that he/she agreed to being called after the trial for an in-depth interview when he/she signed the informed consent form (you can forward it to the participant if required). Right after that, briefly present the study: “We would like to ask you some questions about your experience of this program to help us to think about how it could be improved for delivery in the future. There are no right or wrong answers to the questions we are going to ask. We will be speaking to a number of people that participated in the project, asking everyone the same questions. If you feel unable to answer a question, please say so and we will move on to the next one.”

• “Do you agree to participate? We will be recording the session for transcription purposes. We will erase the tape as soon as we transcribed it. The transcription will be stored securely and will not have any personal data linked to it”

**Overall impressions**

• Could you tell me a little bit about yourself? (E.g., where do you work, what do you fancy, and so on) [Informal, brief first contact]

• Could you tell me how you found out about the program (remember there were two options: remote/ hybrid)?

• How have you found using this stress reduction eHealth intervention?

o What did you like about it?

o What didn’t you like or think could be improved?

o Was it like you expected it to be?

• Did you share with family and/or friends about your involvement in the program? [keep it very short, especially if the person does not engage]

o Explore reasons for sharing/not sharing

o If shared with family: Could you tell more about what you shared about the program with your family/friends/others?

o Explore whether just shared about their participation in the program or also details about the skills learned during the intervention and their experiences of the intervention

**Adaptability**

• How did you find the online/hybrid format?

o How did you feel about having the possibility to choose the format?

• (Online format) Can you describe how you found working with your facilitator?

o Explore acceptability of features of facilitator, i.e. HCWs in training, sometimes working in the same shift, young…

• (Hybrid format) Can you describe how you found attending group sessions with colleagues?

o Explore the advantages/disadvantages of the group influences.

o Explore the advantages/ disadvantages of the face-to-face format.

**Feasibility**

- Do you think is possible to attend this program and practice all day as part of your daily routine?

o Explore if they think the chosen format was the best for them.

- Do you think it is useful for HCWs in primary care?

o If yes, explain.

- Do you think it covers most of the psychological needs of the HCWs?

o If no, explain what would be adequate to complete the intervention program.

**Compatibility**

- Have you felt comfortable taking part in the program?
- Is there other psychological intervention available for HCWs?

If yes, do you think it is compatible with this eHealth intervention?

- Do you think the support program is in line with other self-care beliefs you perceive at work?

Explore both organizational messages and workers’ general beliefs.

- Do you think most HCWs would agree with receiving this support regularly in their workplaces?

**Context**

• Can you describe how easy or difficult you found attending the program/ calls?

o Explore barriers and facilitators to attendance and practice (e.g. timing in the week, recalls; in the hybrid format the role of the group, etc)

o Explore if the facilitator could do/say anything that encouraged attendance or practice.

• Can you identify any factors that motivated you to enroll actively in the program?

• Can you describe how you found implementing the strategies taught to you in your everyday routine?

o Explore each strategy

• Did you continue with the skills you learned as part of the program after the last session?

o Explore reasons for (not) doing so

• [FOR DROP-OUTS ONLY] Can you tell me why you stopped the program?

o Explore reasons through probes (i.e., not having an impact, difficulties in relationship with facilitator/group, other commitments – family/work, etc.)

o Explore possible ways to overcome barriers

o Explore if the online/hybrid format did affect the drop-out

**Culture**

• In your view, do you think there is a need for an intervention like this for HCWs in Madrid? Please explain [advanges over existing mental health services, beneficial only for some, need for different forms of the program, i.e., face-to-face, group, etc.]

• What do you think are the main reasons why HCWs would not choose to participate in a program like this? [time commitment, stigma, e-help, helper’s characteristics, program content]

**Wrap-up**

• Ask the interviewee if they have anything to add. Any additional information is added to the interview notes as required.

• Thank person and leave.

***Interview Script: stakeholders***

***Note.*** We will send a policy brief to all stakeholders prior to the interview. During the interview, we will use this brief to introduce ourselves and to present the research design and the main outcomes.

**Overall impression**

- What do you think about the study? (Prompt questions: was it timely?)
- What do you think about the stress reduction eHealth intervention DWM?

**Adaptability**

- What do you think about the delivery format?

o Explore opinions about the online format

o Explore opinions about the hybrid format

o Do you think it is timely to offer both possibilities?

**Acceptability**

- What do you think about the content of the program? (Prompt questions: what do you think about the exercises? What is your opinion about mindfulness-based practices and emotion regulation skills?
- In your opinion, was the recruitment strategy adequate?
- What is your opinion about the delivery strategy? (ask about format and intervention provider)

o Are they adequate for primary care centers?

**Feasibility**

- Would it be possible to provide it regularly among all HCWs in primary care centres?

o Explore real possibilities for establishing it in the long term.

**Compatibility**

- In your opinion, are decision-makers interested in mental health?
- Are they interested to provide mental health support to their HCWs?

**Culture, complexity and costs:**

- Do you think there is a need for an intervention like this to be provided on a larger scale in SERMAS?

o Explore possible advantages over existing mental health services and the need for achieving higher coverage of treatment for psychological problems

- We are interested to know more about scaling up a stress reduction eHealth intervention. By scaling up we mean making it accessible to all HCWs in Madrid by integrating it into the health system. Based on what you’ve just heard/learned about the program, where in the system in Madrid could you see the intervention being provided? Please explain [multiple options possible].

o In which settings within the SERMAS?

o To which specific groups (e.g., nurses, PC doctors, etc.)

o Who do you think should provide it?

o Could you think of something similar? If yes, can it be integrated in there?

o Explore how can it be funded in the long-term (e.g. health insurance, (local) government, private or donor funds)

- What do you think are the main obstacles for scaling it up in Madrid [refer to possible pathways/systems discussed in previous question; and discuss barriers for each]?

o Explore possible obstacles through probes like the ones below. Not all probes need to be covered; better to select probes based on background/expertise of person being interviewed and those not yet covered in interviews with similar interviewees.

o Structure (‘ways of organizing’): e.g. how to ensure sufficient and sustainable human and financial resources to scale up the program; who could provide it (someone within SERMAS or outside SERMAS); how to ensure physical access for all potential users (e.g. time/costs acceptable, any differences rural/urban and men/women); is there sufficient political will; how to upkeep effectiveness of the intervention during implementation.

o Practice (‘ways of doing’): e.g. how can the intervention be made part of existing care, how to manage training and supervision of helpers, how to prevent burden to helpers and supervisors

o Culture (‘ways of thinking’): e.g. how can we ensure stigma surrounding mental health and help-seeking will not be a major obstacle during scale-up; how can we ensure that non-professional or less-trained providers will be accepted by other health professionals; is there sufficient need/demand for and how can we best reach the target group(s)

- Thank you for explaining some of the obstacles. I am interested to hear your thoughts on what is needed to overcome these obstacles (facilitators); could you tell me more about this?

o Explore ways to overcome the obstacles mentioned in previous question

**Context**

- Finally, do you know of any wider trends that have happened or are current happening in Madrid, which could positively or negatively influence the scaling up this intervention?

o Ask about possible political developments, economic developments, socio-cultural climate, stigma, etc.

o Ask about the possible influence of pandemics like COVID-19

**Self-efficacy**

- Do you think, there are enough resources (economics and human resources) within the SERMAS to deliver and maintain this intervention through time?
- How do you think quality control and accountability can be ensured when scaling-up the stress reduction eHealth intervention?

o Explore what can be done when there is spontaneous scaling-up/mushrooming and how then to ensure the quality and safety of the intervention.

**Wrap-up**

- Ask the interviewee if they have anything to add. Any additional information is added to the interview notes as required.
- Thank person and leave.

1. **StaRI checklist**

| **Checklist item** | | **Reported on page #** | **Implementation strategy** | **Reported on page #** | **Intervention** |
| --- | --- | --- | --- | --- | --- |
|  | |  | **“Implementation strategy” refers to how the intervention was implemented** |  | **“Intervention” refers to the healthcare or public health intervention that is being implemented.** |
| **Title and abstract** | | | | | |
| Title | **1** | 1 | Identification as an implementation study, and description of the methodology in the title and/or keywords | | |
| Abstract | **2** | 2-3 | Identification as an implementation study, including a description of the implementation strategy to be tested, the evidence-based intervention being implemented, and defining the key implementation and health outcomes. | | |
| **Introduction** | | | | | |
| Introduction | **3** | 4 | Description of the problem, challenge or deficiency in healthcare or public health that the intervention being implemented aims to address. | | |
| Rationale | **4** | 5 | The scientific background and rationale for the implementation strategy (including any underpinning theory/framework/model, how it is expected to achieve its effects and any pilot work). | 5 | The scientific background and rationale for the intervention being implemented (including evidence about its effectiveness and how it is expected to achieve its effects). |
| Aims and objectives | **5** |  | The aims of the study, differentiating between implementation objectives and any intervention objectives. | | |
| **Methods: description** | | | | | |
| Design | **6** | 6-7 | The design and key features of the evaluation, (cross referencing to any appropriate methodology reporting standards) and any changes to study protocol, with reasons | | |
| Context | **7** | 6 | The context in which the intervention was implemented. (Consider social, economic, policy, healthcare, organisational barriers and facilitators that might influence implementation elsewhere). | | |
| Targeted ‘sites’ | **8** | 6 | The characteristics of the targeted ‘site(s)’ (e.g locations/personnel/resources etc.) for implementation and any eligibility criteria. | 6 | The population targeted by the intervention and any eligibility criteria. |
| Description | **9** | 7 | A description of the implementation strategy | 6-7 | A description of the intervention |
| Sub-groups | **10** | - | Any sub-groups recruited for additional research tasks, and/or nested studies are described | | |
| **Methods: evaluation** | | | | | |
| Outcomes | **11** | 8 | Defined pre-specified primary and other outcome(s) of the implementation strategy, and how they were assessed. Document any pre-determined targets | - | Defined pre-specified primary and other outcome(s) of the intervention (if assessed), and how they were assessed. Document any pre-determined targets |
| Process evaluation | **12** | 8 | Process evaluation objectives and outcomes related to the mechanism by which the strategy is expected to work | | |
| Economic evaluation | **13** | - | Methods for resource use, costs, economic outcomes and analysis for the implementation strategy | - | Methods for resource use, costs, economic outcomes and analysis for the intervention |
| Sample size | **14** | 6 | Rationale for sample sizes (including sample size calculations, budgetary constraints, practical considerations, data saturation, as appropriate) | | |
| Analysis | **15** | 8 | Methods of analysis (with reasons for that choice) | | |
| Sub-group analyses | **16** | 7-8 | Any a priori sub-group analyses (e.g. between different sites in a multicentre study, different clinical or demographic populations), and sub-groups recruited to specific nested research tasks | | |
| **Results** | | | | | |
| Characteristics | **17** | 9 | Proportion recruited and characteristics of the recipient population for the implementation strategy | 9 | Proportion recruited and characteristics (if appropriate) of the recipient population for the intervention |
| Outcomes | **18** | 9-14 | Primary and other outcome(s) of the implementation strategy | - | Primary and other outcome(s) of the Intervention (if assessed) |
| Process outcomes | **19** |  | Process data related to the implementation strategy mapped to the mechanism by which the strategy is expected to work | | |
| Economic evaluation | **20** | - | Resource use, costs, economic outcomes and analysis for the implementation strategy | - | Resource use, costs, economic outcomes and analysis for the intervention |
| Sub-group analyses | **21** | 9 | Representativeness and outcomes of subgroups including those recruited to specific research tasks | | |
| Fidelity/ adaptation | **22** | 9 | Fidelity to implementation strategy as planned and adaptation to suit context and preferences | 12 | Fidelity to delivering the core components of intervention (where measured) |
| Contextual changes | **23** | 12-14 | Contextual changes (if any) which may have affected outcomes | | |
| Harms | **24** | - | All important harms or unintended effects in each group | | |
| **Discussion** | | | | | |
| Structured discussion | **25** | 14-19 | Summary of findings, strengths and limitations, comparisons with other studies, conclusions and implications | | |
| Implications | **26** | 17-18 | Discussion of policy, practice and/or research implications of the implementation strategy (specifically including scalability) | 18 | Discussion of policy, practice and/or research implications of the intervention (specifically including sustainability) |
| **General** | | | | | |
| Statements | **27** | 6 | Include statement(s) on regulatory approvals (including, as appropriate, ethical approval, confidential use of routine data, governance approval), trial/study registration (availability of protocol), funding and conflicts of interest | | |
